# Retrospective Analysis of the Efficacy and Safety of Ultrasound-Guided Radiofrequency Ablation in the Treatment of Papillary Thyroid Microcarcinoma: A Follow-Up Study of Continuous Postoperative Surveillance and Large-Sample Data

**DOI:** 10.1155/2024/2704087

**Published:** 2024-03-06

**Authors:** Gongli Zhou, Xuefeng Zhang, Ke Xu, Beibei Zhang, Ruiqing Su, Tingting Cai, Wei Zhao, Feng Li

**Affiliations:** Hangzhou Weja Hospital, Hangzhou, China

## Abstract

**Objective:**

To retrospectively evaluate the efficacy and safety of ultrasound-guided radiofrequency ablation (RFA) in the treatment of papillary thyroid microcarcinoma (PTMC) through a follow-up study of continuous postoperative surveillance and large-sample data.

**Methods:**

The efficacy of ultrasound-guided RFA was evaluated by measuring the tumor volume reduction rate (VRR), tumor disappearance rate, and disease progression in 358 patients with low-risk unifocal PTMC who underwent ultrasound-guided RFA at Hangzhou Weja Hospital, while the safety was evaluated by measuring their complications.

**Results:**

The VRR was −745.69 ± 1012.69 (%), −150.35 ± 395.5 (%), 46.47 ± 138.74 (%), 92.95 ± 27.88 (%), 97.78 ± 10.99 (%), and 99.27 ± 3.82 (%), respectively, at 1, 3, 6, 12, 18, and 24 months after RFA. The corresponding tumor disappearance rate was 1.68%, 9.78%, 43.85%, 82.68%, 92.59%, and 95.63%, respectively. No local recurrence, new tumors, lymph node metastasis, distant metastasis, and deaths caused by recurrent/metastatic PTMC were found at the last follow-up. Except for 3 cases (0.84%) with thickening or hoarseness of voice and 3 cases (0.84%) with coughing during drinking water, no other complications were found.

**Conclusions:**

Ultrasound-guided RFA has good efficacy and safety for the treatment of low-risk unifocal PTMC and can be promoted for use in patients who meet the required indications.

## 1. Introduction

Thyroid cancer is the most common endocrine malignancy, of which the detection rate has been increasing year by year [1]. Within 10 years, its proportion in systemic malignancies increased from around 1% to 3.1% [2], accounting for approximately 7%–15% of all thyroid tumors [3]. From the perspective of histopathology, thyroid cancer can be divided into the following four types: papillary thyroid carcinoma (PTC), anaplastic thyroid carcinoma (ATC), follicular thyroid carcinoma (FTC), and medullary thyroid carcinoma (MTC). Among them, PTC is the most common subtype, and its incidence rate ranked first among the four types of thyroid cancer [4, 5], accounting for about 85% of thyroid cancer cases [6, 7]. Among all PTCs, lesions with the longest diameter ≤10 mm are defined by the World Health Organization as papillary thyroid microcarcinoma (PTMC) [8–10]. With the optimization of biopsy technology, development of high-frequency ultrasound, widespread application of ultrasound-guided fine needle aspiration (FNA), widespread popularity of ultrasound-guided thyroid examination, and enhancement of people's awareness of physical examination, PTMCs have shown an upward trend in the number of detected cases [11, 12], accounting for about 50%–87% of all PTCs [13–16]. Although PTMC is highly prevalent, its prognosis is good, with an extremely low mortality rate and a 10-year survival rate far exceeding 90% [1, 10, 17–19]. National Health Commission of the People's Republic of China stated that the mortality rate of PTMC without extrathyroidal extension was “zero” [20].

At present, immediate surgery is the first-line choice for the treatment strategy of PTMC. The overall 10-year survival rate of PTMC patients after thyroidectomy was 94.6%, and the overall 15-year survival rate was 90.7% [21, 22]. However, scholars found that in sharp contrast to the increase in the incidence rate of PTMCs, its mortality remained stable or decreased [14, 23, 24] and also noted that thyroidectomy in all cases may lead to overtreatment [25]. Therefore, the initial treatment of low-risk PTMC has shifted towards more conservative treatment strategies, such as active surveillance or ultrasound-guided thermal ablation. For some qualified patients, these conservative treatments can serve as alternative surgical options [26, 27].

In recent years, with the development of nonsurgical, minimally invasive technology, thermal ablation techniques including radiofrequency ablation (RFA), microwave ablation (MWA), and laser ablation (LA) have been increasingly used in the treatment of benign thyroid nodules and attracted widespread attention. The safety and effectiveness of ultrasound-guided thermal ablation for the treatment of benign thyroid nodules have been confirmed in a great number of clinical studies [28–32]. According to a meta-analysis reported in 2019, the results of thermal ablation were similar with or slightly better than those of active surveillance [33]. In recent years, joint consensuses made by multiple associations and authoritative guidelines from many countries proposed thermal ablation as an alternative to active surveillance or immediate surgery for the treatment of PTMC patients who do not meet surgical conditions or refuse to undergo surgery [6, 26, 34–37]. Although a number of guidelines and consensuses recognized the role of thermal ablation in the treatment of PTMC, it was not recommended as a routine treatment for PTMC but can only be used as a surgical alternative for certain patients meeting the required indications.

Ultrasound-guided RFA has long been widely used in tumor intervention or preliminary treatment for liver, kidney, lung, and other parts of the body. In addition, some studies have shown that RFA has good therapeutic effects on PTMC [38–40]. However, there are still few long-term follow-up studies on the prognosis, safety, and efficacy of RFA for PTMC. The objective of this study was to retrospectively evaluate the efficacy and safety of RFA for low-risk PTMC through a follow-up study of continuous postoperative surveillance and large-sample data. The efficacy of ultrasound-guided RFA was mainly evaluated by measuring the VRR, tumor disappearance rate, and disease progression, and the safety was evaluated by measuring the complications.

## 2. Materials and Methods

This retrospective study was approved by the Ethics Committee of Hangzhou Weja Hospital. We reviewed medical records of the PTMC patients who underwent RFA or surgery from March 2019 to October 2022. Written informed consent was obtained from each patient before a treatment procedure.

### 2.1. Patients

This study was conducted by Hangzhou Weja Hospital. The patients were selected from patients with thyroid disease who underwent ultrasound-guided RFA at Hangzhou Weja Hospital from March 2019 to October 2022. The inclusion criteria were as follows: (1) patients with an appropriate route for FNA revealed by ultrasound; (2) patients with classic PTC confirmed by ultrasound-guided FNA; (3) patients with unifocal PTMC within the thyroid gland with the longest diameter ≤10 mm; (4) patients who had anxiety about PTMC, refused clinical observation, and were unwilling to undergo surgical treatment but hoped to receive ablation treatment; (5) patients who accepted ≥12-month follow-up after RFA; and (6) patients who agreed that their PTMC-related data could be used anonymously for clinical research. The exclusion criteria were as follows: (1) patients with evidence of extrathyroidal extension or capsule contact/invasion; (2) patients with evidence of lymph node metastasis; (3) patients with evidence of distant metastasis; (4) patients with dysfunction or failure of important organs (e.g., brain, heart, liver, and kidney); and (5) patients with severe coagulation mechanism disorders. After screening according to these inclusion and exclusion criteria, a total of 358 patients with low-risk unifocal PTMC were included in this study.

### 2.2. Instruments and Equipment

Voko color Doppler ultrasound diagnostic instrument and L741 high-frequency linear-array probe were used, with the probe frequency set to 12 MHz; MedSphere radiofrequency therapy instrument (produced by MedSphere International, Inc.) was selected, with the frequency set to 400 KHz. In consideration of the small diameters of all PTMCs, we chose type L-121 disposable radiofrequency needles among the different types of RFA needles.

### 2.3. Pre-RFA Assessment

Before RFA, ultrasound was used to evaluate the three orthogonal diameters (i.e., the longest diameter and the other two vertical diameters), position, shape (height/width), contour, echo, calcification, internal structure, vascular density, and adjacent anatomical relationships of each tumor. In addition, coagulation function and routine thyroid function tests were performed on each patient. Before RFA, the treatment methods, procedures, precautions, and potential complications were explained to each patient in detail, and an informed consent was signed by each patient.

### 2.4. RFA Procedure

**Figure 1:**
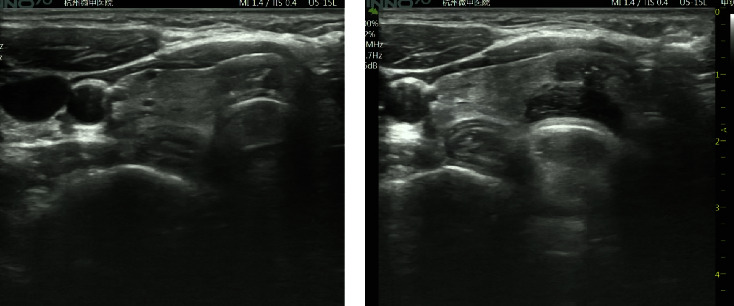
Ultrasound images before and after injection of hydrodissection: (a) image of the tumor before injection of hydrodissection and (b) image of the tumor after injection of hydrodissection.

RFA was performed by experienced surgeons with over 10 years of experience in clinical work. The patient was required to adopt a supine position. The shoulders were appropriately raised to make the head tilt back, and routine disinfection and draping were performed after the neck was completely exposed. 2% lidocaine injection was injected under ultrasound guidance for local anesthesia treatment. Hydrodissection was determined to be used or not based on the positional relationship between the lesion and adjacent tissues and organs. For lesions close to the carotid artery, trachea, esophagus, and recurrent laryngeal nerve, appropriate amount of hydrodissection (e.g., normal saline, 5% glucose, and sodium hyaluronate gel) was injected to form a “hydrodissection zone” (Figure 1) so as to prevent important tissue structures of the neck from being thermally damaged. After the output power of the radiofrequency therapy instrument was adjusted to 15 W, an 18 G radiofrequency needle was inserted deep into the patient's lesion under ultrasound guidance, and the switch was turned on to start treatment. In the following process, moving shot technique via the isthmus was adopted. To avoid tumor residue or recurrence, expanded ablation was used, with the coverage range of per echoic changes exceeding the tumor boundary by more than 3 mm. The treatment ended after per echoic changes completely surrounded the lesion and surrounding area. CEUS examination was performed immediately after RFA to evaluate the completeness of RFA. If there were any residual lesions, supplementary RFA was performed immediately. After the completion of RFA, the patient was arranged to stay in the treatment room for 1-2 hour observation. Their physical conditions were closely monitored and their complications during and after RFA were evaluated. After another 3–5 hour continuous observation in the hospital, those without any abnormalities were discharged.

### 2.5. Follow-Up after RFA

**Figure 2:**
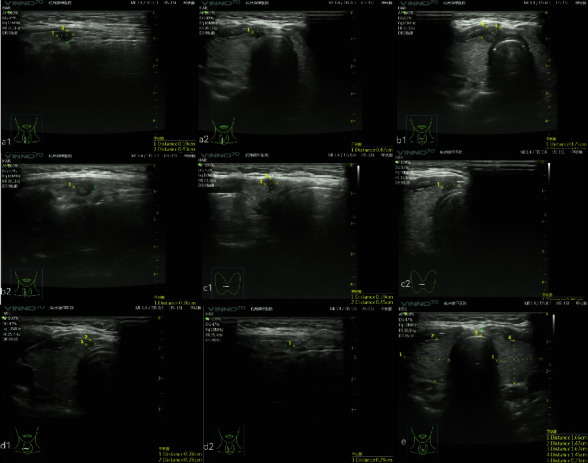
Ultrasound images before and after RFA: (a1-a2) the tumor had a volume of 55.83 mm^3^ before RFA; (b1-b2) the ablated tissue had a volume of 164.27 mm^3^ at 1 month after RFA; (c1-c2) the volume of the ablated tissue decreased to 36.78 mm^3^ at 3 months after RFA; (d1-d2) the volume of the ablated tissue decreased to 11.91 mm^3^ at 6 months after RFA; and (e) the tumor was completely absorbed at 12 months after RFA.

The following indicators were observed at 1, 3, 6, 12, 18, and 24 months after RFA and every 6–12 months thereafter based on ultrasound evaluation and relevant data collected during the follow-up: (1) tumor volume (V), as shown in Figure 2, calculated using the formula: V (mm^3^) = abc *π*/6 [41–43] (*a* is the longest diameter, while *b* and *c* are the other two diameters perpendicular to *a*); (2) tumor volume reduction rate (VRR), calculated as VRR = (initial volume of the tumor—follow-up volume of the ablated tissue)/initial volume of the tumor X 100% [39, 44]; (3) local recurrence or new occurrence of tumors and lymph node metastasis or distant metastasis evaluated through thyroid and neck lymph node ultrasound and ultrasound-guided FNA of suspicious nodules; and (4) fatality of recurrent or metastatic PTMC.

### 2.6. Statistical Processing

Statistical analysis was conducted using SPSS software (SPSS for Windows 21.0). Quantitative data that conformed to normal distribution and homogeneity of variance were expressed as the mean ± standard deviation. Count data were expressed in percentages and frequencies. Comparison of volume before and after RFA was performed using the paired *t*-test, and differences with *P* < 0.05 were considered statistically significant.

## 3. Results

### 3.1. Baseline Characteristics

According to the inclusion and exclusion criteria set in this study, a total of 358 patients with low-risk unifocal PTMC who underwent ultrasound-guided RFA at Hangzhou Weja Hospital from March 2019 to October 2022 were included in this study, with a follow-up period of not less than 12 months. The baseline characteristics of the cases included are shown in Table 1, including 67 men (18.72%) and 291 women (81.28%); 303 cases (84.64%) were under 55 years old, and 55 cases (15.36%) were 55 years old or above; 153 cases (42.74%) had the longest tumor diameter ≤5 mm, and 205 cases (57.26%) had the longest tumor diameter greater than 5 mm but less than or equal to 10 mm; 127 cases (35.47%) had an aspect ratio less than 1, and 231 cases (64.53%) had an aspect ratio greater than or equal to 1; 159 tumors (44.41%) were located in the left lobe of the thyroid gland, 162 (45.25%) were located in the right lobe of the thyroid gland, and 37 (10.34%) were located in the isthmus; 146 cases (40.78%) had calcification, and 212 cases (59.22%) had no calcification.

### 3.2. RFA and Follow-Up Data

According to Table 2, the mean age of the 358 patients with unifocal PTMC was 43.37 ± 11.22 years, with the maximum age being 75 and the minimum age being 18. The mean longest diameter of the tumor before RFA was 5.71 ± 1.89 mm, with a maximum of 10 mm and a minimum of 2 mm. The mean tumor volume before RFA was 78.5 ± 78.04 mm, with a maximum of 471.6 mm^3^ and a minimum of 3.14 mm^3^. The mean RFA time was 126.99 ± 131.65 seconds, with a maximum of 1200 seconds and a minimum of 15 seconds. The mean RFA power was 22.81 ± 9.63 w, with a maximum of 50 w and a minimum of 9 w. The mean follow-up period after RFA was 22.81 ± 9.63 months, with a maximum follow-up period of 56 months and a minimum follow-up period of 14 months. Among them, 270 cases had a follow-up period of not less than 18 months, and 206 cases had a follow-up period of not less than 24 months.

### 3.3. Tumor Changes after RFA

According to Table 3, the volume of ablated tissue was 0–2023.44 (389.24 ± 331.19) mm^3^ at 1 month after RFA and 0–903.1 (113.83 ± 152.66) mm^3^ at 3 months after RFA, both significantly larger than the initial volume before RFA (78.5 ± 78.04 mm^3^), with the difference being statistically significant (*P* < 0.001). The reason of larger volume should be attributed to the use of expanded ablation during RFA to achieve the goal of eliminating the lesion. Starting from the 6^th^ month, the volume of ablated tissue was significantly smaller than the initial volume, and the differences were statistically significant (*P* < 0.001). Specifically, the volume of ablated tissue was 0–775.83 (31.27 ± 72.03) mm^3^ at 6 months after RFA, 0–276.33 (4.61 ± 22.2) mm^3^ at 12 months after ablation, 0–133.25 (1.76 ± 11.67) mm^3^ at 18 month after RFA, and 0–9.46 (0.28 ± 1.36) mm^3^ at 24 months after RFA.

Since the volume of ablated tissue at 1 and 3 months after RFA was still larger than the initial volume before RFA, the corresponding VRR was negative, −745.69 ± 1012.69 (%) and −150.35 ± 395.5 (%), respectively. Starting from the 6th month, the VRR turned positive and gradually increased; it was 46.47 ± 138.74 (%) at 6 months after RFA; at 12 months after RFA, the VRR was found to have a significant increase, reaching 92.95 ± 27.88 (%); and at 18 months after RFA, the VRR was 97.78 ± 10.99 (%); at 24 months after RFA, the VRR was 99.27 ± 3.82 (%). There were 6 cases (1.68%) where the tumor completely disappeared at 1 months after RFA, 35 cases (9.78%) where the tumor completely disappeared at 3 months after FRA, 157 cases (43.85%) where the tumor completely disappeared at 6 months after RFA, and 296 cases (82.68) where the tumor completely disappeared at 12 months after RFA; among the 270 patients followed up for not less than 18 months, 250 cases (92.59%) had their tumors completely disappearing at 18 months after RFA; among the 206 patients followed up for not less than 24 months, 197 cases (95.63%) had their tumors completely disappearing at 24 months after RFA.

### 3.4. Disease Progression after RFA

Table 4 shows ultrasound-displayed disease progression after RFA. It can be seen that there was no evidence of disease progression after RFA in the 358 patients with unifocal PTMC included in this study within 12 months. Among the 270 patients followed up for not less than 18 months, no local recurrence, new tumors, lymph node metastasis, distant metastasis, and deaths caused by recurrent/metastatic PTMC were found at the last follow-up.

### 3.5. Complications of RFA

Hydrodissection functions in protecting important tissue structures, especially the recurrent laryngeal nerve, trachea, esophagus, and carotid artery, from heat damage. Therefore, the use of hydrodissection is related to complications occurring during and after RFA.  Table 5 evaluates the use of hydrodissection and complications. According to Table 5, hydrodissection was used in a total of 341 cases (95.25%); normal saline was used in 13 cases with diabetes (3.63%) as recommended in “expert consensus on thermal ablation for thyroid benign nodes, microcarcinoma, and metastatic cervical lymph nodes (2018 edition)” [34]; 5% glucose was used in 277 cases (77.37%); sodium hyaluronate gel was used in 4 cases (1.12%); and 5% glucose and sodium hyaluronate gel was used in 47 cases (13.13%). Except for 3 cases (0.84%) with thickening or hoarseness of voice and 3 cases (0.84%) with coughing during drinking water, there were no serious complications such as dysphagia, bleeding, or infection, permanent recurrent laryngeal nerve injury, tracheal or esophageal injury, thyroid dysfunction, and parathyroid gland injury. The 3 patients with thickening or hoarseness of voice recovered without treatment within three months; the 3 patients with coughing during drinking water recovered without treatment within 1–4 weeks after RFA.

## 4. Discussion

Currently, most guidelines concerning the treatment of thyroid cancer classified thyroidectomy as the first-line treatment strategy for low-risk unifocal PTMC [42, 45, 46]. Although PTMC without lymph node metastasis is a low-risk type of cancer, traditional or endoscopic thyroidectomy is still the main treatment method [47]. However, there are constant controversies among scholars regarding the treatment strategies for low-risk PTMC [48–50], given that thyroidectomy often causes serious harm to patients, including damage to important adjacent structures such as the recurrent laryngeal nerve and parathyroid gland, leads to various mild or severe complications, scar residue, and cosmetic trouble, requires lifelong medication, affects the quality of life of patients to some extent, and requires a relatively high cost of resection [51–54]. A Finnish autopsy report showed that occult papillary carcinoma (≤5 mm) was a normal finding and should not be blindly surgically removed [55]. Subsequently, many scholars raised objections to the adoption of immediate thyroidectomy for all low-risk PTMCs [56, 57].

Considering that PTMC is featured with good prognosis, low invasiveness, and low risk of metastasis, some studies suggested active surveillance as the first-line strategy for the management of PTMC [58–60]. Active surveillance was initiated by Kuma Hospital in Japan in 1993 as a PTMC-targeted management method, and in a series of subsequent studies, the reliability and safety of active surveillance replacing immediate surgery have been reasonably and tenably demonstrated [59, 61–63]. Especially in a 30-year follow-up study, Kuma Hospital found that out of 3222 patients who accepted active surveillance, only 124 (3.8%) had an increase of 3 mm in the longest tumor diameter, with the tumor growth rate being only 4.7% and 6.6%, respectively, at 10 and 20 years [63]. In 2015, the American Thyroid Association proposed that active surveillance could be an alternative to immediate surgery for PTMCs [42]. However, “living with cancer” means that patients still face the possibility of lymph node metastasis and tumor progression, which affects their quality of life and psychological state, and most people have poor compliance in terms of accepting follow-up [33]. Due to the absence of reliable clinical or imaging methods to identify the small portion of invasive PTMCs, many patients suffer from high-level anxiety during active surveillance and follow-up. Among the patients who met the indications for active surveillance, 8.7–32% ultimately turned to surgery without clear evidence of the need for immediate surgical intervention [33]. Thermal ablation, as a treatment method for PTMC that can significantly alleviate patient anxiety and potentially reduce the risk of tumor progression or metastasis, is expected to become a parallel option for active surveillance.

According to the expert consensus released jointly by Chinese Medical Doctor Association and other relevant academic organizations, thermal ablation can be used to treat some metastatic lymph nodes of thyroid cancer under strict requirements on indications [34]. For thyroid patients who are not qualified for surgery or refuse surgery, Korean Society of Thyroid Radiology proposed in its 2017 guideline that thermal ablation can be implemented before tumor progression [35]. In view of the advantages of thermal ablation including safety, effectiveness, precise positioning, and simple operation, Chinese Ultra Sound Doctors Association recommended inactivating tumors through the use of ablation to alleviate patient anxiety [36]. The guideline jointly released by European Thyroid Association and Cardiovascular and Interventional Radiological Society of Europe in 2021 included PTMC as one of the indications for thermal ablation [26]. The international multidisciplinary consensus statement jointly written by multiple organizations including British Association of Endocrine and Thyroid Surgeons also pointed out that thermal ablation can be used for the treatment of unifocal PTMC [37].

Applied in the treatment of thyroid diseases, RFA has been found to have many advantages [26, 38–41], such as easy and simple operation, high repeatability, strong controllability, less trauma, fewer complications, faster postoperative recovery, better preservation of thyroid function, reduction of patient pain, and improvement of patient quality of life. The effectiveness and safety of RFA in the treatment of benign thyroid tumors have been verified in a large number of studies [64–66], and it has been rapidly promoted as the first-line treatment method for nonmalignant thyroid nodules. The principle of RFA lies in the release of thermal energy towards the target tissue through the electrode, thereby damaging the tumor tissue which is ultimately absorbed by the body [67].

The effectiveness of RFA in the treatment of low-risk PTMC has also been verified. General Hospital of Chinese PLA has found in a series of studies that RFA can effectively eliminate low-risk PTMCs [68–71], and in a 5-year follow-up study, it was found that the efficacy of RFA was not inferior to surgery [72]. Especially in a 24–69 months' follow-up study of 414 patients with low-risk unifocal PTMC, General Hospital of Chinese PLA found that the VRR was 98.81 ± 6.41%, and the tumor disappearance rate was 88.41% at the last follow-up [69]. The 928^th^ Hospital of the PLA Joint Logistics Support Force conducted a 12–18 months' follow-up on 214 patients with low-risk unifocal PTMC, and found that the VRR was 99.9 ± 0.2% at 18 months and that there were no local recurrence, new tumors, or distant metastasis during the follow-up period and no serious complications [73]. Their VRR result at 18 months was higher than the result of this study (97.78 ± 10.99%) and the research result from General Hospital of Chinese PLA because they excluded tumors with the presence of calcification. Although some calcified tumors included in this study were not fully absorbed at 24 months, all cases were found to have a significant decrease in volume of ablated tissue compared to the initial volume, showing that the goal of slowing down tumor progression or outward metastasis was reached. In a more than five years' follow-up study of 74 patients who underwent RFA in South Korea, it was found that the tumor disappearance rate reached 98.8% at 24 months and 100% at 60 months, where only 3 patients were found to have new tumors, which disappeared completely after another RFA and no local tumor progression, lymph node metastasis, distant metastasis, or surgical delays were found during the follow-up period [41]. In a literature review in 2021, 13 clinical studies (1389 patients and 1422 tumors) were included, and it was found that during a mean follow-up period of 7.8–72 months, the VRR was 98.5–100 and the tumor disappearance rate was 33.7–100% [74]. In a meta-analysis of RFA and surgery in 2022, 8 studies involving a total of 1932 PTMC patients were included, where it was found that compared with the thyroidectomy group, the RFA group had fewer complications after RFA, shorter operation time, less intraoperative blood loss, and shorter postoperative hospital stay and that the incidence of postoperative recurrence or metastasis was similar between the two groups [75]. Zhao and Song found that the total incidence of postoperative complications was 25.49% in the thyroidectomy group and 1.96% in the RFA group [76]. In two comparative studies of RFA and thyroidectomy procedures, it was found that there was no significant difference in local progression, incidence of complications, and lymph node metastasis between RFA and thyroidectomy procedures, but RFA resulted in shorter surgical and hospitalization time, lower costs, and higher quality of life, and no disabling complications were reported in the RFA group, while there were a total of 7 permanent complications in the thyroidectomy group (2 cases of permanent recurrent laryngeal nerve injury and 5 cases of permanent hypoparathyroidism) [72, 77]. This study also confirmed the efficacy of RFA from the perspectives of VRR, tumor disappearance rate, and disease progression and verified the safety of RFA from the perspectives of complications. It can be seen that RFA is equivalent in efficacy and superior in safety to thyroidectomy.

However, there are some limitations in this study. First of all, this study is a single-center retrospective analysis. Therefore, further evidence should be collected from multicenter prospective studies to support the conclusions of this study. Second, we only evaluated the results of RFA treating low-risk unifocal PTMC but did examine the efficacy and safety of RFA in treating multifocal PTMC. Third, we did not collect our own samples to compare the efficacy between surgery and RFA in the treatment of low-risk unifocal PTMC. Fourth, since no surgery was performed after RFA among our patients up to now, it is not possible for us to evaluate whether RFA increases the difficulty and risk of subsequent surgical procedures. Fifth, it is still necessary to observe the progression of disease after RFA in a longer time.

In conclusion, traditional thyroidectomy, active surveillance, and thermal ablation are the main interventions for low-risk PTMC. They have their own advantages and disadvantages and are complementary to each other. The selection of an optimal treatment requires the patient to participate actively and the clinical doctor to evaluate all factors. Active surveillance effectively avoids overtreatment but faces great difficulties in practice due to the patient's fear of cancer and poor in terms of accepting the follow-up. For patients with low-risk unifocal PTMC who have a strong desire to seek active treatment and meet the indications for thermal ablation, ultrasound-guided RFA may be a better choice, as a large amount of clinical results and the large-sample data of this study have confirmed that RFA, when performed properly, can effectively reduce tumor volume (most noncalcified tumors have been absorbed by the body after RFA), slow down disease progression, avoid the occurrence of serious complications, eliminate anxiety in patients, preserve their thyroid function, and improve their quality of life.

## Figures and Tables

**Table 1:** Baseline characteristics of 358 patients with unifocal PTMC.

Characteristics	Cases, *n* = 358 (%)
Gender	
Male	67 (18.72)
Female	291 (81.28)
Age	
<55	303 (84.64)
≥55	55 (15.36)
Longest diameter (mm)	
≤5	153 (42.74)
5.1–8.9	178 (49.72)
9-10	27 (7.54)
Aspect ratio	
<1	127 (35.47)
≥1	231 (64.53)
Location	
Left lobe	159 (44.41)
Right lobe	162 (45.25)
Isthmus	37 (10.34)
Calcification	
With calcification	146 (40.78)
No calcification	212 (59.22)

**Table 2:** RFA and follow-up data of the 358 patients with unifocal PTMC.

Items	Max	Min	Mean ± SD
Age (years)	75	18	43.37 ± 11.22
Longest diameter before RFA (mm)	10	2	5.71 ± 1.89
Tumor volume before RFA (mm^3^)	471.6	3.14	78.5 ± 78.04
RFA time (s)	1200	15	126.99 ± 131.65
RFA power (w)	50	9	22.81 ± 9.63
Follow-up period (m)	56	14	27.7 ± 10.54

**Table 3:** Tumor volume, VRR, and tumor disappearance after RFA in the 358 patients with unifocal PTMC.

Follow-up period	Tumor volume			*P*	VRR (%)			Disappearance	
	Max (mm^3^)	Min (mm^3^)	Mean ± SD		Max	Min	Mean ± SD	Cases	Rate (%)
1 month	2023.44	0	389.24 ± 331.19	<0.001	100	−8576.77	−745.69 ± 1012.69	6/358	1.68
3 months	903.1	0	113.83 ± 152.66	<0.001	100	−3021.68	−150.35 ± 395.5	35/358	9.78
6 months	775.83	0	31.27 ± 72.03	<0.001	100	−1542.52	46.47 ± 138.74	157/358	43.85
12 months	276.33	0	4.61 ± 22.2	<0.001	100	−133.28	92.95 ± 27.88	296/358	82.68
18 months	133.25	0	1.76 ± 11.67	<0.001	100	15.95	97.78 ± 10.99	250/270	92.59
24 months	9.46	0	0.28 ± 1.36	<0.001	100	68.23	99.27 ± 3.82	197/206	95.63

Note. *P* was a statistical result of the comparison between the volume of ablated tissue at each follow-up period and the initial volume before RFA.

**Table 4:** Disease progression after RFA in the 358 patients with unifocal PTMC.

Follow-up period	Local recurrence	New tumor	Lymph node metastasis	Distant metastasis	Death caused by PTMC
1 month	0	0	0	0	0
3 months	0	0	0	0	0
6 months	0	0	0	0	0
12 months	0	0	0	0	0
18 months	0	0	0	0	0
24 months	0	0	0	0	0

**Table 5:** Use of hydrodissection and complications in the 358 patients with unifocal PTMC.

Items	Cases, *n* = 358 (%)
Hydrodissection	341 (95.25)
Normal saline	13 (3.63)
5% glucose	277 (77.37)
Sodium hyaluronate gel	4 (1.12)
5% glucose and sodium hyaluronate gel	47 (13.13)
No	17 (4.75)
Complication	6 (1.68)
Thickening or hoarseness of voice	3 (0.84)
Dysphagia	0 (0)
Coughing during drinking water	3 (0.84)
Bleeding or infection	0 (0)
Permanent recurrent laryngeal nerve injury	0 (0)
Tracheal or esophageal injury	0 (0)
Thyroid dysfunction	0 (0)
Parathyroid injury	0 (0)

## Data Availability

The output data used to support the findings of this study are included within the article. The detailed patient data used to support the findings of this study are restricted by the Ethics Committee of Hangzhou Weja Hospital in order to protect PATIENT PRIVACY. Data are available from Gongli Zhou (zgongli@163.com) for researchers who meet the criteria for access to confidential data.
